# Suppression of STAT3 and HIF-1 Alpha Mediates Anti-Angiogenic Activity of Betulinic Acid in Hypoxic PC-3 Prostate Cancer Cells

**DOI:** 10.1371/journal.pone.0021492

**Published:** 2011-06-24

**Authors:** Jimin Shin, Hyo-Jeong Lee, Deok-Beom Jung, Ji Hoon Jung, Hyo-Jung Lee, Eun-Ok Lee, Seok Geun Lee, Beom Sang Shim, Seung Hoon Choi, Seong Gyu Ko, Kwang Seok Ahn, Soo-Jin Jeong, Sung-Hoon Kim

**Affiliations:** College of Oriental Medicine, Kyung Hee University, Seoul, South Korea; McMaster University, Canada

## Abstract

**Background:**

Signal transducer and activator of transcription 3 (STAT3) is a transcription factor that regulates various cellular processes such as cell survival, angiogenesis and proliferation. In the present study, we examined that betulinic acid (BA), a triterpene from the bark of white birch, had the inhibitory effects on hypoxia-mediated activation of STAT3 in androgen independent human prostate cancer PC-3 cells.

**Methodology/Principal Findings:**

BA inhibited the protein expression and the transcriptional activities of hypoxia-inducible factor-1α (HIF-1α) under hypoxic condition. Consistently, BA blocked hypoxia-induced phosphorylation, DNA binding activity and nuclear accumulation of STAT3. In addition, BA significantly reduced cellular and secreted levels of vascular endothelial growth factor (VEGF), a critical angiogenic factor and a target gene of STAT3 induced under hypoxia. Furthermore, BA prevented *in vitro* capillary tube formation in human umbilical vein endothelial cells (HUVECs) maintained in conditioned medium of hypoxic PC-3 cells, implying anti-angiogenic activity of BA under hypoxic condition. Of note, chromatin immunoprecipitation (ChiP) assay revealed that BA inhibited binding of HIF-1α and STAT3 to VEGF promoter. Furthermore, silencing STAT3 using siRNA transfection effectively enhanced the reduced VEGF production induced by BA treatment under hypoxia.

**Conclusions/Significance:**

Taken together, our results suggest that BA has anti-angiogenic activity by disturbing the binding of HIF-1α and STAT3 to the VEGF promoter in hypoxic PC-3 cells.

## Introduction

Signal transducer and activator of transcription 3 (STAT3) is one of STAT protein family and constitutively active in a wide range of human cancer cells [1]. Activated STAT3 proteins by cytokines and growth factors form homo- or heterodimers, and then translocate from the cytoplasm to the nucleus, where they are binding to the promoter of various gene products involved in anti-apoptosis (bcl-2, bcl-x_L_ and survivin), proliferation (cyclin D1), and angiogenesis (vascular endothelial growth factor (VEGF)) [2]. Interestingly, recent studies reported that STAT3 is activated in response to hypoxia, a common feature of various solid tumors [3], [4]. Activated STAT3 mediates the up-regulation of hypoxia inducible factor alpha (HIF-1α), a major regulator to adapt under hypoxic conditions by increasing its stability and transcriptional activity [5]. Thus, recently STAT3 and HIF-1α are attractive target molecules by natural compounds and herbal extracts in cancer research.

Betulinic acid (BA), initially reported as a human melanoma-specific inhibitor, is a triterpenoid mainly derived from the bark of the white birch (*Betula pubescens*) [6]. Recent evidences suggest the anti-cancer effects of BA [7], [8], anti-inflammatory [9] and anti-viral [10] activities via various signaling pathways such as epidermal growth factor receptor (EGFR) [11], hedgehog [12], signal transducer and activator of transcription 3 (STAT3) [13] and nuclear factor-kappa B (NF-κB) [14]. Nonetheless, there is no evidence that BA mediates anti-cancer activity through inhibiting STAT3 signaling in solid tumors.

Thus, in the present study, we investigated the roles of STAT3 and HIF-1 α in BA induced anti-angiogenic activity in hypoxic PC-3 prostate cancer cells by MTT assay, Western blotting, immunocytochemistry, ELISA and EMSA.

## Results

### Cytotoxic effect of betulinic acid (BA) against PC-3 cells

**Figure 1:**
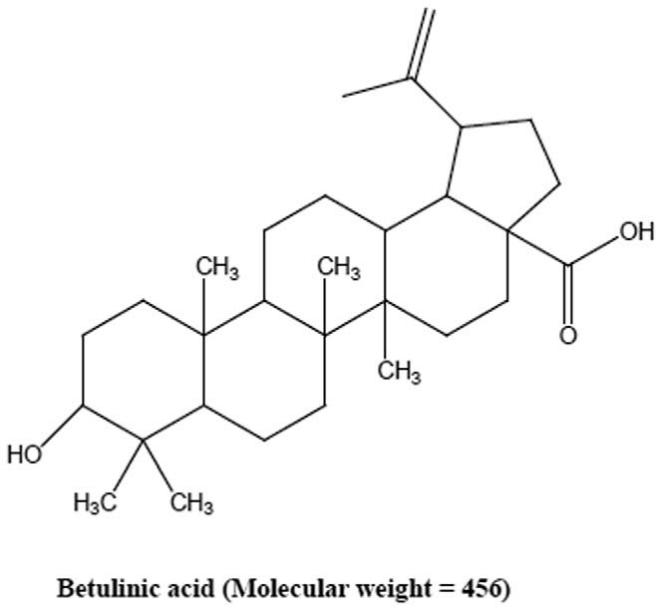
Chemical structure of betulinic acid (BA). Molecular weight  =  456.

**Figure 2:**
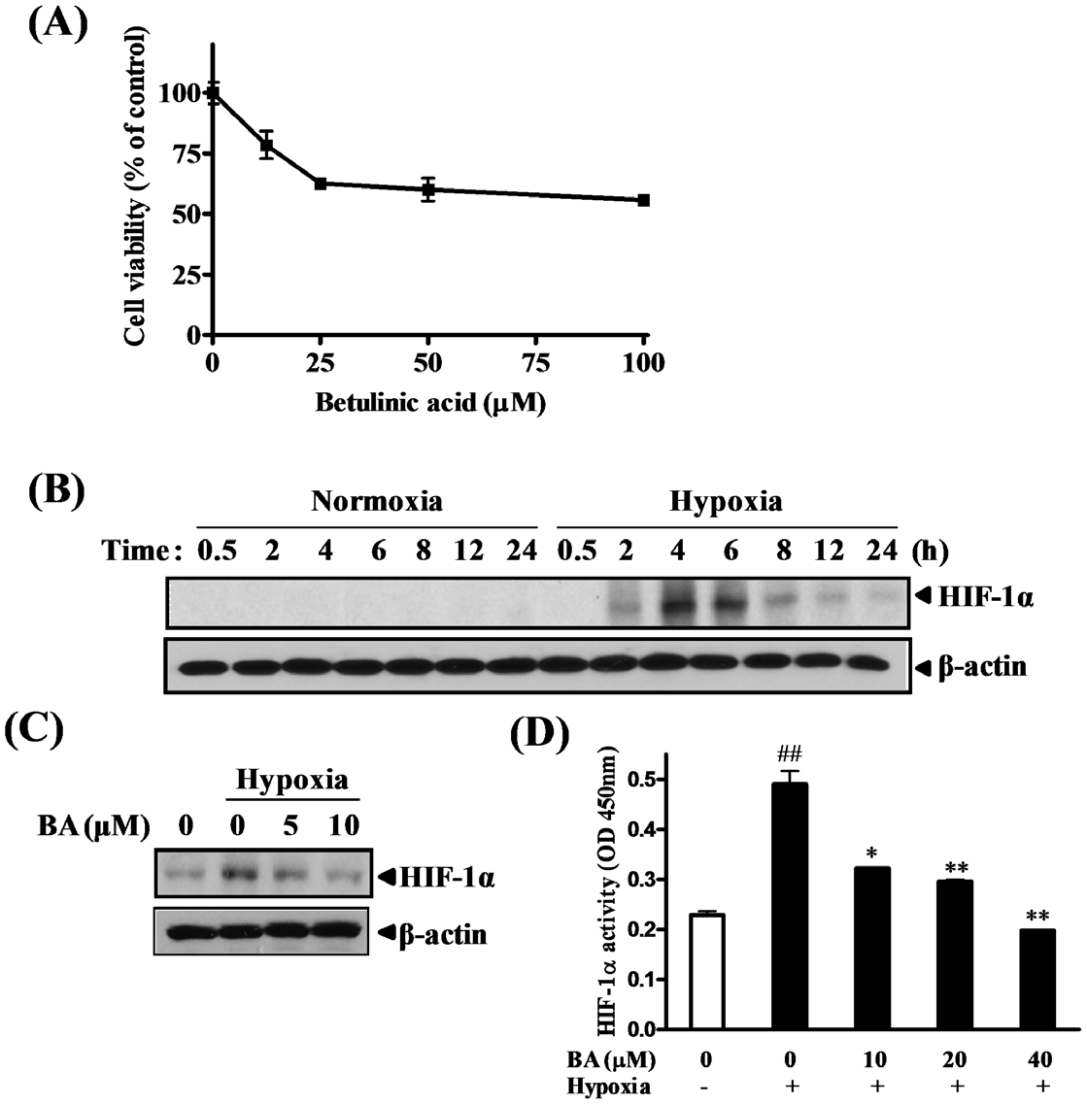
Effect of betulinic acid (BA) on hypoxia-induced HIF-1α activation in PC-3 cells. (A) PC-3 cells were treated with various concentrations of BA (0, 12.5, 25, 50 or 100 µM) for 24 h. Cell viability was analyzed by MTT assay. (B) Cells were exposed to normoxia or hypoxia for 0.5, 2, 4, 6, 8, 12 or 24 h. Cell lysates were prepared and subjected to Western blotting to determine the expression of HIF-1α. (C) Cells were treated with or without BA (5 or 10 µM) under normoxic or hypoxic condition for 4 h. Cell lysates were prepared and subjected to Western blotting to determine the expression of HIF-1α. (D) Nuclear extract was prepared from the cells treated with BA (0, 10, 20 or 40 µM) under normoxia or hypoxia for 4 h. HIF-1α transcription activity was measured by using TransAM HIF-1 transcription factor assay kit. Data represent means ± S.D. ^##^, p<0.01 *vs* normoxia control, and ^*^, p<0.05 and ^**^ < 0.01 *vs* hypoxia control.

Cytotoxic effect of BA (Fig. 1) was evaluated by MTT assay. PC-3 cells were treated with various concentrations of BA (0, 12.5, 25, 50 or 100 µM) for 24 h. Cell viability was reduced to 78.49±5.67 and 62.64±1.26% at concentrations of 12.5 and 25 µM, respectively, and sustained to∼60% at over 25 µM (Fig. 2A).

### Effect of betulinic acid (BA) on hypoxia-induced HIF-1α activation in PC-3 cells

Hypoxia is a hallmark of solid tumors [15] and HIF-1α is a transcription factor that responses to hypoxia [16]. To examine whether BA can affect HIF-1α induced by hypoxia, we first determined the best time point of hypoxia-induced HIF-1α expression in PC-3 cells. Cells were exposed to normoxia or hypoxia for 0.5, 2, 4, 6, 8, 12 or 24 h. HIF-1α expression was dramatically induced under hypoxic condition for 4 h (Fig. 2B). Then, cells were treated with or without BA under hypoxia for 4 h. BA decreased hypoxia-induced HIF-1α expression in a dose-dependent manner compared with hypoxia control (Fig. 2C). In addition, hypoxia significantly activated HIF-1α transcription while BA treatment inhibited the hypoxia-mediated transcriptional activation of HIF-1α in a dose-dependent manner (Fig. 2D). These results suggest that BA has the ability to inhibit the expression as well as transcription of HIF-1α in hypoxic PC-3 cells.

### Effect of betulinic acid (BA) on hypoxia-induced STAT3 activation in PC-3 cells

**Figure 3:**
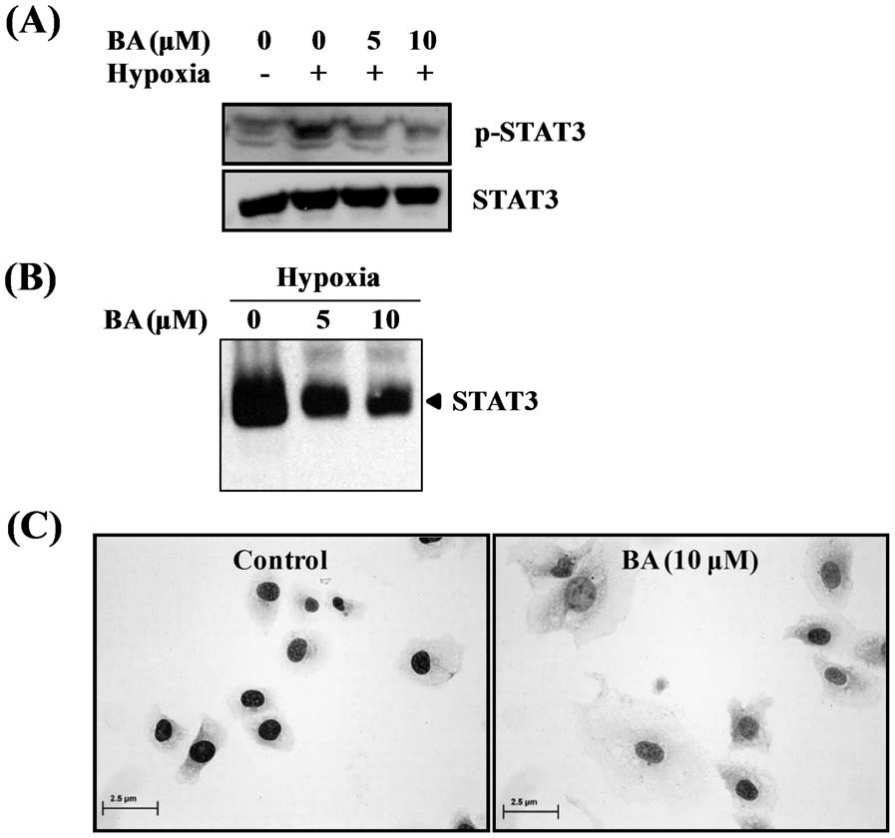
Effect of betulinic acid (BA) on hypoxia-induced STAT3 activation in PC-3 cells. PC-3 cells were treated with or without BA (5 or 10 µM) under normoxic or hypoxic condition for 4 h. (A) Cell lysates were prepared and subjected to Western blotting for phospho-STAT3 and STAT3. (B) Nuclear extracts were prepared and applied to EMSA to analyze the STAT3-DNA binding activity. (C) Cells were treated with or without BA (10 µM) under hypoxia. Immunocytochemistry was performed for STAT3. DAB (brown) and hematoxylin-eosin was used as a substrate and a counterstaining, respectively.

Recent studies reported that a transcription factor STAT3 is involved in the transcriptional regulation of HIF-1α [17]. In our study, hypoxia enhanced phospho-STAT3 level while normoxia did not affect it. BA treatment inhibited hypoxia-mediated STAT3 phosphorylation in a dose-dependent manner (Fig. 3A). Also, EMSA revealed that BA prevented the STAT3/DNA binding activity under hypoxia in a dose-dependent manner (Fig. 3B). Furthermore, imunocytochemical (ICC) staining with anti-HIF-1α antibody showed a significant nuclear expression of HIF-1α under hypoxic condition. In contrast, BA treatment attenuated HIF-1α expression in the nucleus in hypoxic PC-3 cells (Fig. 3C), suggesting its inhibitory effect on the nuclear translocation of HIF-1α.

### Effects of betulinic acid (BA) on hypoxia-induced angiogenesis

**Figure 4:**
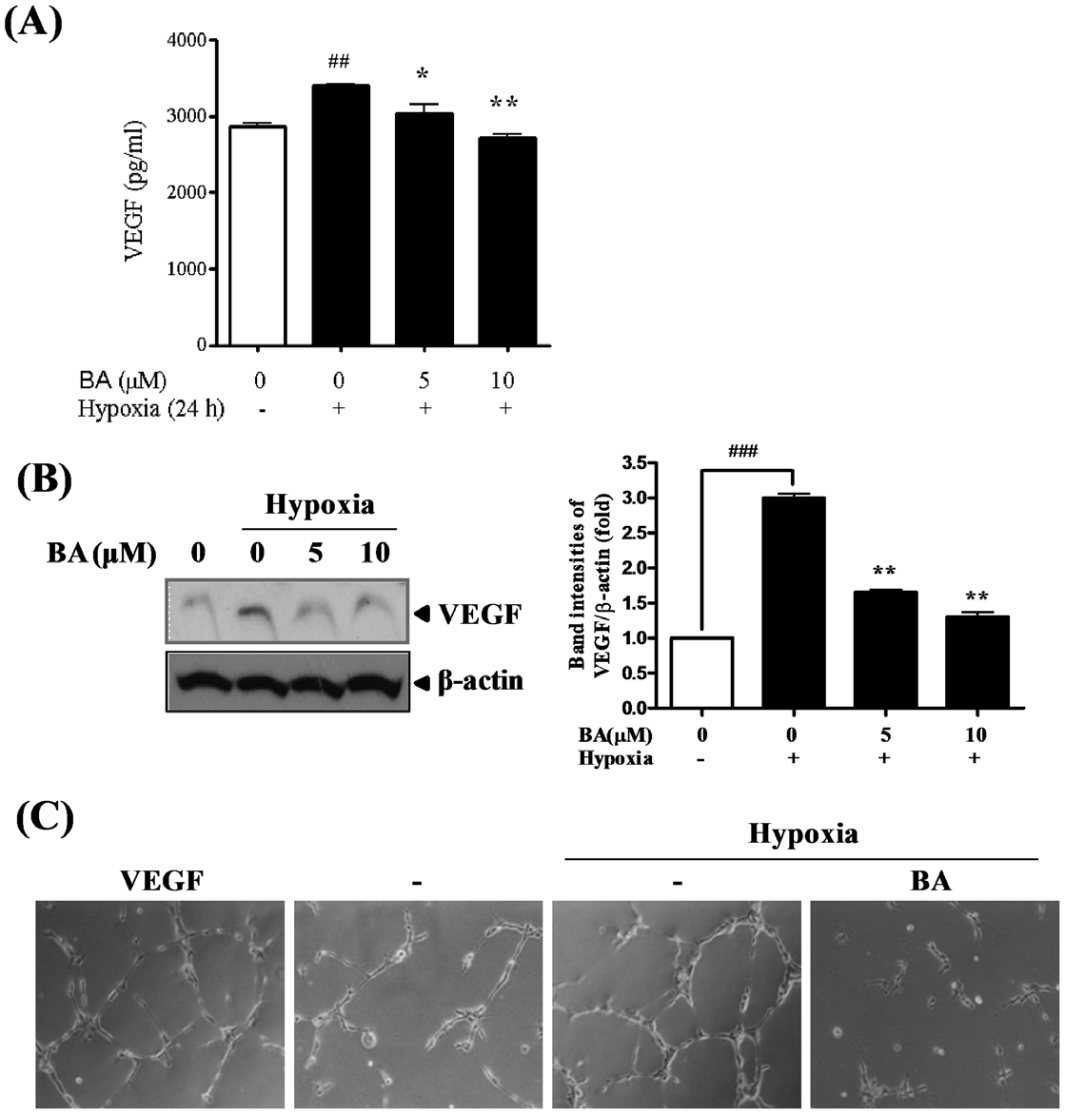
Effect of betulinic acid (BA) on hypoxia-induced angiogenesis. (A and B) PC-3 cells were treated with 0, 5 or 10 µM BA for 24 h. (A) VEGF levels in the culture supernatants were measured by using a Quantikine VEGF ELISA kit. (B) Cell lysates were prepared and subjected to Western blotting to determine VEGF expression. Graphs represent relative band intensities of VEGF/β-actin. Data represent means ± S.D. ^##^, p<0.01 *vs* normoxia control, and ^*^, p<0.05 and ^**^ <0.01 *vs* hypoxia control. (C) HUVECs were treated with VEGF (20 ng/ml) as positive control or the culture supernatant from PC-3 cells treated with or without BA (10 µM) under normoxia or hypoxia. Tube formation assay was performed using growth factor reduced Matrigel. Cells were fixed with Diff-Quick solution, photographed randomly under an Axiovert S 100 light microscope at ×100 magnification and counted.

Hypoxia is one of angiogenesis inducers through HIF-1α activation [18]. Thus, the inhibitory effect of BA was evaluated on hypoxia-mediated angiogenesis. VEGF, a critical angiogenesis factor [19], was evaluated at the secreted cellular and protein levels by ELISA and Western blotting, respectively. BA significantly reduced VEGF production in a dose-dependent manner by ELISA (Fig. 4A). Consistently, BA attenuated VEGF protein expression in a dose-dependent manner by Western blotting (Fig. 4B).

Additionally, HUVEC tube formation assay, which is known as a typical angiogenesis *in vitro* model, was performed to confirm anti-angiogenic effect of BA on hypoxia-mediated angiogenesis. VEGF was used as a positive control of angiogenesis induction. HUVECs mixed with the supernatants from PC-3 cells were cultured in the absence or presence of BA under hypoxia. As shown in Fig. 4C, hypoxia-induced tube formation was prevented by BA treatment in PC-3 cells while clear tube formation was exhibited in untreated control under hypoxia, suggesting that BA inhibits hypoxia-mediated angiogenesis.

### Effects of betulinic acid (BA) on the binding of STAT3 and HIF1 α to VEGF promoter in hypoxic PC-3 cells

**Figure 5:**
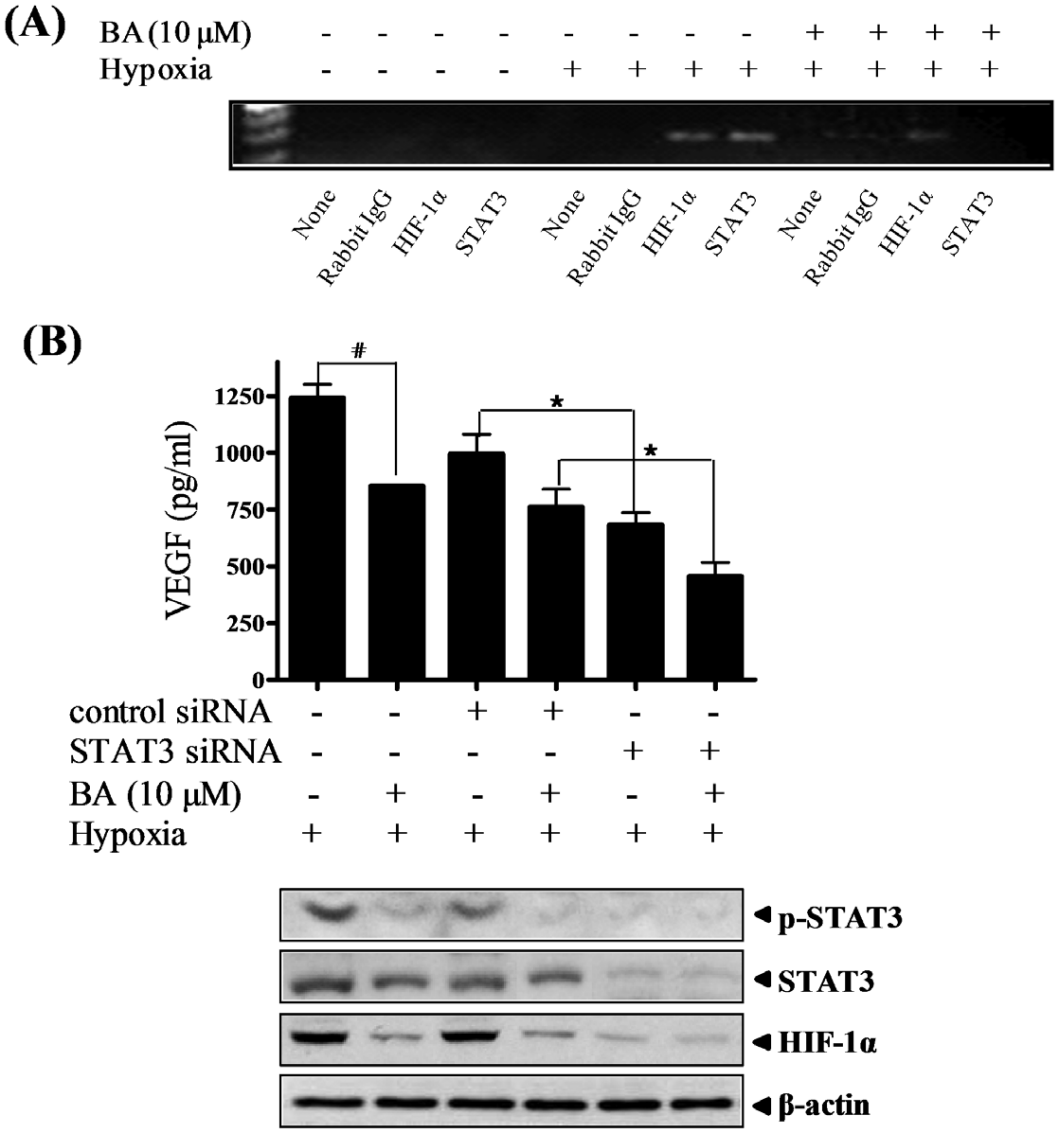
Effect of betulinic acid (BA) on STAT3 binding on the VEGF promoter in hypoxic PC-3 cells. (A) PC-3 cells were treated with or without BA (10 µM) under normoxia or hypoxia for 4 h. The immunoprecipitated DNA with rabbit normal IgG, HIF-1α or STAT3 antibody was amplified by PCR analysis for VEGF promoter. (B) Cells were transiently transfected with siRNA for scramble or STAT3 for 24 h and treated with or without BA (10 µM) for 18 h under hypoxia. VEGF levels in the culture supernatants were measured by using a Quantikine VEGF ELISA kit. Data represent means ± S.D. ^#^, p<0.05 *vs* control, and ^*^, p<0.05 *vs* control siRNA. Cell lysates were subjected to Western blotting for phospho-STAT3, STAT3 and HIF-1α.

Recent studies revealed that STAT3 activation is directly link to the transcriptional regulation of VEGF by binding to the VEGF promoter [20], [21]. In light of this event, we conducted chromatin immunoprecipitation (ChiP) assay. As shown in Fig. 5A, the binding activity of STAT3 and HIF-1α to the VEGF promoter was detected under hypoxia (lanes 5-8) compared with normoxia (lanes 1-4). Notably, BA treatment suppressed the binding of STAT3 and HIF-1α to VEGF promoter in hypoxic condition (lanes 9-12).

In order to confirm the critical role of STAT3 in anti-angiogenic regulation of BA in hypoxic PC-3 cells, STAT3 siRNA transfection was carried out in PC-3 cells. Treatment with either BA or STAT3 siRNA reduced the production of VEGF by 39.6% and 45.9% respectively, compared with untreated control. Furthermore, BA treatment significantly reduced VEGF production by 63.25% in STAT3 siRNA-transfected PC-3 cells (Fig. 5B). Western blotting revealed that siRNA for STAT3, but not control, efficiently blocked STAT3 (Fig. 5B).

## Discussion

Prostate cancer classified as an adenocarcinoma is the second most common malignant tumors in American men, with estimates of 192,280 new cases and approximately 27,360 deaths in 2009 [22], [23]. Betulinic acid (BA), a plant-derived pentacyclic lupane-type triterpenoid, can be extracted from various plants such as *Sarracenia flava* [24], *Diospyros* spp., *Inga punctata* [25], *Ziziphus* spp., and *Vauquelinia corymbosa* [26]. Several groups reported anti-cancer activity of BA in various cancers including lung, colorectal, breast, prostate and cervical cancer [27], but not normal cells [28]. Also, BA completely inhibited tumour growth without toxicity in athymic mice bearing human melanomas [6]. Moreover, anti-cancer activity of BA was exerted by inducing apoptosis in the cancer cells. For example, BA-induced apoptosis was independent of p53 in neurorectodermal tumor [29] and melanoma cells [30]. In neuroblastoma cells, BA induced apoptosis through loss of the mitochondrial membrane potential, reactive oxygen species (ROS) production and caspase activation [31].

Interestingly, Karna and colleagues recently reported that BA inhibited the expression of HIF-1α and vascular endothelial growth factor (VEGF) in human endometrial cancer cells [32]. However, the regulatory mechanisms whereby BA inhibits angiogenesis are not fully understood. In the present study, we found that BA suppressed hypoxia-mediated protein accumulation, transcriptional activation and nuclear localization of HIF-1α in PC-3 cells. Consistent with the results of Karna's paper, our data also showed that BA significantly inhibited VEGF secretion and protein expression in hypoxic PC-3 cells. Additionally, *in vitro* tube formation assay further confirmed anti-angiogenenic effect of BA in hypoxic PC-3 cells.

Recently, Niu and colleagues suggested that constitutively activated STAT3 up-regulated VEGF and induced tumor angiogenesis [20]. Also, Wei and colleagues reported that STAT3 activation regulates the expression of VEGF and human pancreatic cancer angiogenesis Furthermore, several papers described the role of STAT3 as a potential modulator of HIF-1α-induced VEGF signaling in cancer cells [4], [33]. In this regard, the effect of BA on STAT3 and HIF-1α activation was examined in hypoxic PC-3 cells in our study. Consistent with the evidences by Pandey and colleagues that BA suppressed STAT3 activation in multiple myeloma cells [13], BA prevented hypoxia-induced tyrosine phosphorylation, DNA binding activity and nuclear translocalization of STAT3, suggesting the inhibitory effect of BA on STAT3 activation.

VEGF promoter contains various transcription factor binding sites including STAT3 [20] as well as HIF-1 [34]. Physical interaction of STAT3 with HIF-1 controls VEGF transcriptional activation by their binding to the VEGF promoter [4]. In our study, hypoxia promoted the binding of STAT3 and HIF-1α to the VEGF promoter in PC-3 cells. In contrast, BA remarkably inhibited the binding of STAT3 and HIF-1α to the VEGF promoter site under hypoxic condition. Additionally, silencing STAT3 using its specific siRNA significantly enhanced BA-mediated inhibition of VEGF production, implying the involvement of STAT3 in anti-angiogenic regulation of BA in hypoxic PC-3 cells. Similar to our study, Gariboldi and colleagues reported that NVP-AEW541, a IGFR1 inhibitor, disrupted IGF/STAT3/HIF1 pathway in human glioblastoma cells [35]. Leeman-Neill and colleagues also reported that Guggulsterone inhibited STAT3 and HIF-1α and suggested a biologic rationale for further clinical investigation BA for human head and neck squamous cell carcinoma (HNSCC) therapy [36].

Collectively, our data demonstrate that BA suppressed expression and transactivation of hypoxia-induced HIF-1α, STAT3, VEGF as well as capillary tube formation in PC-3 cells. It is noteworthy that anti-cancer activity of BA is exerted by inhibiting angiogenesis via inhibition of binding of STAT3 and HIF-1α to the VEGF promoter in PC-3 cells. Thus, our findings suggest that BA can be a potent anti-angiogenic agent by targeting STAT3/HIF-1α/VEGF signaling for prostate cancer therapy.

## Materials and Methods

### Compounds

Betulinic acid (BA) (Figure 1) was purchased from Sigma-Aldrich (St. Louis, MO) and dissolved in dimethyl sulfoxide (DMSO) as a 10 mM stock solution for experimental use.

### Cell culture

Human prostate cancer cell line PC-3 was obtained from American Type Culture Collection (ATCC, Rockville, MD) and maintained in RPMI1640 (Welgene, Daegu, Korea) supplemented with 10% fetal bovine serum (FBS) and 1% antibiotic-antimyotic solution. Human umbilical vein endothelial cells (HUVECs) were isolated from fresh human umbilical cord vein and maintained in EBM-2 (Lonza, Valais, Switzerland) supplemented with 2% FBS, 0.04% hydrocortisone, 0.1% VEGF, 0.1% IGF-1, 0.4% hFGF-B, 0.1% hEGF, 0.1% ascorbic acid, and 1% heparin.

### Hypoxia induction

Cells were incubated in anaerobic incubator at 94% N_2_, 5% CO_2_ and 1% O_2_ (Thermo scientific, Rockford, IL) as previously described [37].

### Cytotoxicity assay

To evaluate cytotoxicity of BA, 3-(4,5-dimethylthiazol-2-yl)-2,5-diphenyltetrazolium bromide (MTT) assay was performed as previously described [38]. PC-3 cells were plated onto 96-well microplates at a density of 1×10^4^ cells per well and exposed to various concentrations of BA (0, 12.5, 25, 50 or 100 µM) for 24 h. MTT solution (1 mg/ml) (Sigma-Aldrich) was added onto each well and incubated for 2 h at 37°C. Extraction buffer (20% SDS and 50% dimethylformamide) was then added and optical density (OD) was measured using microplate reader (Tecan Austria GmbH, Grödig, Austria) at 570 nm. Cell viability was calculated as a percentage of viable cells in BA-treated group versus untreated control by following equation.

Cell viability (%)  =  [OD (BA) - OD (Blank)] / [OD (Control) - OD (Blank)] ×100

### Western blot analysis

Whole-cell extracts were prepared using lysis buffer [50 mM Tris (pH 7.5), 150 mM NaCl, 1% triton X-100, 0.01% SDS, 1 mM EDTA (pH 8.0) and protease inhibitor cocktail tablets (Roche Applied Science, Inndianapolis, IN)]. Nuclear and cytoplasmic extracts were obtained by fractionated by using NE-PER nuclear and cytoplasmic extraction reagents (Thermo scientific, Rockford, IL). Protein samples were separated on 10% SDS-PAGE gel and transferred to a nitrocellulose membrane. The membrane was blocked in 5% nonfat skim milk, and probed with primary antibodies for HIF-1α (1∶500, Gene Tex, Irvine, CA), STAT3 (1∶1000, Cell Signaling, Danvers, MA), phospho-STAT3 (1∶500, Cell Signaling, Danvers, MA), VEGF (1∶500, Santa Cruz Biotechnologies, Santa Cruz, CA) and β-actin (Sigma, St. Louis, MO) overnight at 4°C. The membranes were exposed to HRP-conjugated secondary antibodies for 2 h at room temperature and protein expression was detected by using enhanced chemiluminescence (ECL) Western blotting detection reagent (GE Health Care Bio-Sciences, Piscataway, NJ).

### HIF-1α transcription activity assay

HIF-1α transcriptional activity was analyzed by HIF-1α transcription factor assay using TransAM HIF-1 transcription factor assay kit (Active Motif, Carlsbad, CA) according to the manufacturer's instructions. Briefly, nuclear extracts were added onto 96-well microplate coated with oligonucleotides containing hypoxia response element (HRE) (5′-TACGTGCT-3′) from the erythropoietin (EPO) gene. HIF dimers present in nuclear extracts bind with high specificity to this response element and are subsequently detected with an antibody directed against HIF-1α. Addition of a secondary antibody conjugated to horseradish peroxidase (HRP) provides a sensitive colorimetric readout that is easily quantified by spectrophotometry. Values are expressed as optical density (OD) at 450 nm with a reference wavelength of 655 nm.

### Immunocytochemistry

PC-3 cells were seeded on 4-chamber slides at a density of 3×10^4^ cells per chamber and treated either with or without BA (10 µM) under hypoxia as previously described [37]. The cells were fixed in 4% formaldehyde solution for 10 min at room temperature and blocked in blocking buffer (10% BSA/Triton X-100 in PBS) containing 6% horse serum for 1 h at room temperature. The slides were incubated with anti-STAT3 (1:100) antibody overnight at 4°C and then probed with anti-mouse or rabbit biotinylated antibodies (Vector Labs, Burlingame, CA) for 1.5 h at room temperature. The expression was detected by using Vector ABC complex/HRP kit (Vector Labs, Burlingame, CA) and color-developed with 3,3′-diaminobenzidine tetrahydrochloride in dark. The specimens were then counterstained with hematoxylin-eosin (Sigma-Aldrich, St. Louis, MO) and analyzed under a microscope (Leica Microsystems Res., Wetzlar, Germany).

### Electrophoretic mobility shift assay (EMSA)

The STAT3-DNA binding was analyzed by electrophoretic mobility shift assay (EMSA) using Gelshift Chemiluminescent EMSA kit (Active Motif, Carlsbad, CA) as previously described [39]. Briefly, nuclear extracts were prepared from anethole-treated cells and incubated with STAT3 consensus oligonucleotides (5′-CTT CAT TTC CCG TAA ATC CCT AAA GCT-3′) (Santa Cruz Biotechnologies, Santa Cruz, CA). The DNA-protein complex formed was separated from free oligonucleotides on 5% native polyacrylamide gels. Chemiluminescent detection was performed using ECL reagents according to the vendor's protocols (GE Health Care Bio-Sciences, Piscataway, NJ).

### In vitro tube formation assay

In vitro tube formation assay was performed as previously described [40]. Matrigel (BD) was added on 24-well plates and polymerized by incubating for 1 h at 37°C. HUVECs were seeded onto Matrigel coated plates and incubated in EBM-2 supplemented with VEGF (20 ng/ml) or the supernatant from PC-3 cells treated with BA (0 or 10 µM) under normoxia or hypoxia for 24 h. After 8 h incubation, cells were fixed with 4% formaldehyde and randomly chosen fields were photographed under an Axiovert S 100 light microscope (Carl Zeiss, Weimar, Germany) at 100×magnification.

### Enzyme-linked immunosorbent assay (ELISA) for VEGF

PC-3 cells were plated onto 60-mm dish at a density of 1×10^6^ cells/plate and incubated in the absence or presence of BA (10 µM) under normoxia or hypoxia for 24 h. VEGF level in the supernatant was measured by using human VEGF ELISA kit according to the manufacturer's protocol (Biosource International Inc., Camarillo, CA).

### Chromatin immunoprecipitation (ChiP) assay

PC-3 cells were plated onto 100-mm dishes at a density of 1.5×10^6^ cells/dish, treated with BA for 4 h under normoxic or hypoxic condition and then 1% formaldehyde and 0.125 M glycine. Soluble chromatins were isolated by using EZ-Zyme chromatin prep kit (Millipore, Billerica, MA) and immunoprecipitated with antibodies of normal rabbit IgG (EMD biosciences, Gibbstown, NJ), HIF-1α or STAT3. Histone/DNA crosslinks were reversed by adding 5 M NaCl at 65°C for 4 h, followed by phenol/chloroform extraction and ethanol precipitation. PCR reaction was performed to amplify VEGF promoter using ChiP primers (sense 5′-AGACTCCACAGTGCATACGTG-3′ and antisense 5′-AGTGTGTCCCTCTGACAATG-3′.

### siRNA trasnfection

PC-3 cells were transiently transfected with scramble or STAT3 siRNA (SantaCruz biotechnology, SantaCruz, CA) at 50 nM by using INTERFERin siRNA transfection reagent (Polyplus-transfection Inc., New York, NY). After incubation for 24 h, the cells were treated with BA and maintained for 18 h under hypoxia.

### Statistical analysis

All data were expressed as means ± S.D. Statistical significance was analyzed by student's t-test.
